# Growth pattern of children with congenital heart disease before and after open heart surgery

**DOI:** 10.3389/fped.2025.1463998

**Published:** 2025-07-24

**Authors:** Ahmad Jamei Khosroshahi, Maryam Shoaran, Shamsi Ghaffari, Ehsan Shabanpour, Parmida Seraj Ebrahimi, Akram Ansari, Reza Khosravi, Eftekhar Azarm, Shahram Sadeghvand, Gisou Erabi, Fatemeh Chichagi

**Affiliations:** ^1^ Pediatric Cardiology Department, Pediatric Health Research Center, Tabriz University of Medical Sciences, Tabriz, Iran; ^2^ Pediatric Health Research Center, Tabriz University of Medical Sciences, Tabriz, Iran; ^3^ Student Research Committee, Faculty of Medicine, KTO Karatay University, Konya, Türkiye; ^4^ Shantou University Medical College, Shantou, Guangdong, China; ^5^ Student Research Committee, School of Nursing and Midwifery, Shahid Beheshti University of Medical Sciences, Tehran, Iran; ^6^ School of Medicine, Isfahan University of Medical Sciences, Isfahan, Iran; ^7^ Student Research Committee, Urmia University of Medical Sciences, Urmia, Iran; ^8^ Tehran Heart Center, Tehran University of Medical Sciences, Tehran, Iran

**Keywords:** congenital heart disease, growth patterns, postoperative complications, malnutrition, open heart surgery

## Abstract

**Background:**

Congenital heart disease (CHD) affects 0.8%–1.2% of newborns globally, posing challenges to growth and nutrition. This cross-sectional study, conducted at Shahid Madani Hospital, Tabriz, aimed to assess growth patterns in 200 CHD patients (cyanotic and non-cyanotic) aged 1 month to 15 years before and after open-heart surgery.

**Methods:**

Data were collected from March 2016 to March 2020, including patient records, growth measurements, surgical details, and postoperative outcomes. Patients were categorized into cyanotic and non-cyanotic groups. Inclusion criteria involved CHD patients undergoing surgery with 1–2 years of follow-up. To compare growth disorders between cyanotic and non-cyanotic groups, logistic regression was used, adjusting for age and gender. The significance level was set at 0.05 for statistical analysis.

**Results:**

A total of 200 patients suffered from CHD underwent growth disorder examination in before and after surgery. The prevalence of weight growth disorder reduced from 44% in preoperative to 3.5% in 2 years after surgery. Similarly, the height and head circumference growth disorders experienced reduction from 40.5% and 6.5% to 9.5% and 1.5%, respectively. Generally, growth disorders were higher in cyanotic compared to non-cyanotic group. However, this difference was only significant in weight and height growth disorders in cyanotic vs. non-cyanotic groups after 1 year from surgery (*p* = 0.024, 0.038). The duration of PICU (8.11 ± 5.47 vs. 8.84 ± 5.83 days) and ward (4.40 ± 3.46 vs. 3.57 ± 2.20 days) hospitalization was longer for non-cyanotic vs. cyanotic groups, both suffering from growth disorders.

**Conclusion:**

Despite advancements in pediatric cardiac surgery, CHD children face growth challenges, especially in the cyanotic group. Timely surgical intervention demonstrated improvements, but growth disorders persisted in the long term, impacting PICU and inpatient ward stays. Targeted interventions are crucial to mitigate mortality risks associated with growth impairments in CHD children.

## Introduction

Congenital heart disease (CHD) is the most prevalent abnormality in newborns that affects approximately 0.8%–1.2% of live births worldwide (1). In the modern era, almost 90% of children with critical or complex CHD are anticipated to survive to adulthood, mainly due to advancements in diagnostic technology, surgical management, and postoperative care (2). A physical growth evaluation is frequently used to determine a child's general health and ability to thrive (1). It is generally recognized that children with CHD might experience systemic growth and development difficulties (3). Children with CHD face the dual challenges of malnutrition and growth failure, influenced by cardiac and extracardiac factors. These factors encompass feeding challenges, insufficient caloric intake, inefficient nutrient absorption and utilization, and heightened metabolic demands. The extent and nature of malnutrition may be associated with variables such as the presence of cyanosis, congestive cardiac failure, and pulmonary hypertension (4, 5).

In children with CHD, the severity of malnutrition can range from mild malnutrition to failure to thrive (FTT) (1). The FTT is not a disease by itself but rather a symptom of a more general pathology caused by one or more medical, psychological, or environmental conditions that results in stunted growth in a child. The etiology of failure to thrive in CHD patients is still unknown. Many variables, including calorie intake, malabsorption, increased energy demand, relative hypoxia, and endocrine adaptability, may contribute to such a situation (6). Accompanying hypoxia in patients with cyanosis, increased pulmonary blood flow, and pulmonary hypertension worsen the situation in patients with cyanosis. The hypoxemia caused by the right-to-left shunting of blood flow at the ventricular level in congenital heart disease with cyanotic abnormalities frequently leads to problems with weight gain and height attainment. The left-to-right shunting of blood at the atrial or ventricular level in non-cyanotic congenital heart disease affects weight rather than height (7).

During the initial years of childhood, numerous studies have indicated some catch-up in weight and height following corrective surgery in children with CHD. This catch-up growth typically becomes evident as early as 3 months after the corrective cardiac intervention, extending up to the first year thereafter (8, 9).

Subsequently, as the growth trajectory gradually decelerates and levels off, it signifies the diminishing influence of CHD. Beyond this point, various other factors may come into play, potentially contributing to the enduring presence of malnutrition (9). It is noteworthy that, unlike the impaired growth observed in younger individuals with CHD, studies involving teenagers and young adults reveal a susceptibility to overweight and obesity (10, 11).

The purpose of this study was to enhance the initial outcomes of open-heart surgery in children with CHD by focusing on the timely diagnosis and management of growth and nutrition disorders. Our primary goal was to evaluate the growth patterns in children with cyanotic vs. non-cyanotic CHD before and after surgery.

## Methods

### Study design

This was a cross-sectional study in children aged from 1 month to 15 years with congenital cyanotic and non-cyanotic heart diseases scheduled for cardiac surgery between March 29, 2016, and March 19, 2020, at the Shahid Madani Hospital in Tabriz. Data were collected by reviewing patients’ records, and the information was extracted using a specifically prepared form for children with congenital heart diseases and growth disorders. A total of 200 patients were enrolled in the study, selected through complete random sampling. The study population included 100 patients with congenital cyanotic heart disease (25 patients with tetralogy of Fallot, 25 with pulmonary atresia, 25 with tricuspid atresia, and 25 with single ventricle) and 100 patients with congenital non-cyanotic heart disease (25 with large ventricular septal defect, 25 with large atrial septal defect, 25 with coarctation of the aorta, and 25 with patent ductus arteriosus).

### Inclusion and exclusion criteria

The inclusion criteria were: (1) children with congenital cyanotic and non-cyanotic heart diseases undergoing surgery, (2) absence of other conditions leading to growth disorders, (3) complete and accurate information available in their records, and (4) follow-up for 1–2 years after the operation. The criteria for the exclusion of the study were patients who had underlying diseases that lead to a developmental disorder and the information included in their file was not complete, or they were not followed up during the next 2 years.

### Variable measurement

The collected data included age, diagnosis of congenital heart disease, type of cardiac surgery performed, weight, height, head circumference at birth and the first preoperative visit, duration of cardiopulmonary bypass, duration of stay in the Pediatric Intensive Care Unit (PICU) and ward, postoperative mortality, number of readmissions, postoperative complications, duration of breastfeeding, initiation of complementary feeding, and weight, height, and head circumference at one and 2 years after surgery. This information was obtained through telephone interviews during the specified intervals.

Weight measurements were obtained using a digital scale, while the length of babies was measured by laying them down and using a stretch measuring tape from the top of the head to the bottom of the heel. For children, body height was measured with the child standing against the wall in an upright position, barefoot, hands hanging freely, and eyes looking straight ahead. Additionally, head circumference was measured by placing a tape around the head, passing through the glabella on the forehead and the back of the head (occipital protuberance). All measurements were recorded in centimeters.

### Statistical analysis

In this study, the data were analyzed using SPSS version 26. Normality of the data was assessed using the Kolmogorov–Smirnov test. Descriptive statistics, including percentages for qualitative data and means for quantitative data, were used. To compare growth disorders between cyanotic and non-cyanotic groups, logistic regression was used, adjusting for age and gender. The significance level was set at 0.05 for statistical analysis.

### Ethical issues

This cross-sectional study was approved in accordance with ethical standards by the Tabriz University of Medical Sciences (IR.TBZMED.REC.1400.1188). The required information was retrospectively extracted from patients’ past hospitalization records, ensuring confidentiality and involving no intervention or additional cost to the patients. All procedures adhered to the ethical standards of the responsible committee on human experimentation and the Helsinki Declaration of 1975, as revised in 2008. Data confidentiality was strictly maintained, with access limited to the researchers.

## Results

### Demographic characteristics

In this study conducted in the Cardiac Department of Shahid Madani Hospital in Tabriz over the years 2016–2020, the age range of 200 patients under investigation was 1 month to 15 years, with an average age deviation of 2.6 (±3.4) years. They were categorized into five age groups: 1–5 months, 6–11 months, 12–24 months, 2–4 years, and over 4 years. Regarding gender, there were 105 male individuals (52.5%) and 95 female individuals (47.5%). The majority of patients, comprising 85 individuals (42.5%), were observed in the preoperative visit within the age group of 1–5 months.

### Disease-related characteristics

At the time of birth, 49 individuals (24.5%) among newborns experienced weight growth disorders, with 31 children having cyanotic congenital heart diseases and 18 children with non-cyanotic congenital heart diseases. Additionally, stunted height growth was observed in 22 children with cyanotic diseases and 14 children with non-cyanotic diseases. Nine children with cyanotic diseases and two children with non-cyanotic diseases also suffered from head circumference growth disorders at birth. It is noteworthy that the highest prevalence of weight and height growth disorders at birth occurred in patients diagnosed with Single Ventricle, with 10 children (20.40%) and in those with Aortic Coarctation, with 10 children (16.32%). The highest prevalence of head circumference growth disorders was observed in children diagnosed with Tetralogy of Fallot (TOF), with four children (36.36%).

### Preoperative visit

In preoperative evaluation, weight growth disorder (WGD) was observed in 88 individuals (44%) of the total population of the studied, which was 39 (44.3%) in cyanotic patients and 49 (55.7%) in non-cyanotic patients. Height Growth Disorder (HGD) was seen in 81 (40.5%) of patients, which was higher in the cyanotic group (46, 56.8%) than the non-cyanotic (35, 43.2%). HCGD had the lowest outbreak and affected 13 patients (6.5%) of the total population, with 8 (61.5%) of cases in the cyanotic and 5 (38.5%) in non-cyanotic group (Table 1).

**Table 1:** Prevalence of growth disorders in cyanotic vs. non-cyanotic groups at different time points.

Growth disorder	Time point	Cyanotic (*N* = 100)	Non-cyanotic (*N* = 100)	Total prevalence (%)
Weight (WGD)	Pre-op	39 (44.3%)	49 (55.7%)	88 (44%)
	6-Month post-op	27 (50.0%)	27 (50.0%)	54 (27%)
	1-Year post-op	14 (77.8%)	4 (22.2%)	18 (9%)
	2-Year post-op	6 (85.7%)	1 (14.3%)	7 (3.5%)
Height (HGD)	Pre-op	46 (56.8%)	35 (43.2%)	81 (40.5%)
	6-Month post-op	27 (57.4%)	20 (42.6%)	47 (23.5%)
	1-Year post-op	12 (75.0%)	4 (25.0%)	16 (8%)
	2-Year post-op	12 (63.2%)	7 (36.8%)	19 (9.5%)
Head Circ. (HCGD)	Pre-op	8 (61.5%)	5 (38.5%)	13 (6.5%)
	6-Month post-op	5 (71.4%)	2 (28.6%)	7 (3.5%)
	1-Year post-op	4 (80.0%)	1 (20.0%)	5 (2.5%)
	2-Year post-op	2 (66.7%)	1 (33.3%)	3 (1.5%)

According to the logistic regression model adjusted for age and gender, in the preoperative stage, there were no statistically significant differences in the likelihood of growth disorders between the cyanotic and non-cyanotic groups (Table 2).

**Table 2:** Comparison of growth disorders between cyanotic and non-cyanotic groups at different time points using logistic regression adjusted for age and gender.

Variable	WGD OR (95% CI)	*p*-value	HGD OR (95% CI)	*p*-value	HCGD OR (95% CI)	*p*-value
Group (ref = non-cyanotic)						
Cyanotic	1.15 (0.70–1.89)	.577	0.88 (0.54–1.45)	.638	1.28 (0.45–3.66)	.652
Study visit (ref = pre-op)						
6-Month post-op	0.49 (0.28–0.85)	.011	0.44 (0.24–0.78)	.005	0.37 (0.12–1.09)	.071
1-Year post-op	0.20 (0.09–0.44)	<.001	0.20 (0.08–0.49)	<.001	0.25 (0.08–0.84)	.024
2-Year post-op	0.09 (0.03–0.26)	<.001	0.40 (0.18–0.86)	.019	0.15 (0.04–0.63)	.009
Study visit × group interaction						
6-Month post-op	1.032 (0.538–1.981)	.923	1.167 (0.648–2.102)	.603	1.298 (0.476–3.544)	.538
1-Year post-op	1.795 (1.147–2.808)	.024	1.894 (1.028–3.494)	.038	1.487 (0.754–2.933)	.284
2-Year post-op	1.688 (0.876–3.253)	.109	1.566 (0.775–3.165)	.202	1.203 (0.526–2.749)	.645

The prevalence of patients with growth disorders in the initial preoperative visit, based on the type of cardiac lesion, is detailed in Table 3. Among these, 16 individuals (18.2%) with COA had weight below the 5th percentile, 15 individuals (18.52%) with VSD had height below the 5th percentile, and 3 individuals (23.08%) with Tricuspid atresia had head circumference below the 5th percentile.

**Table 3:** Prevalence of growth disorders in relation to cardiac lesion type at preoperative, 6-month, 1-year, and 2-year postoperative time points.

CHD group	Time point	WGD	HGD	HCGD
Total	Pre-op	88	81	13
	6 Month post-op	54	47	7
	1 Year post-op	18	16	5
	2 Year post-op	7	19	3
Non-cyanotic	Pre-op	49	35	5
	6 Month post-op	27	20	2
	1 Year post-op	4	4	1
	2 Year post-op	1	7	1
Large ASD	Pre-op	11	8	0
	6 Month post-op	8	5	0
	1 Year post-op	2	1	0
	2 Year post-op	0	2	0
Large VSD	Pre-op	14	15	2
	6 Month post-op	6	8	1
	1 Year post-op	2	2	1
	2 Year post-op	1	0	1
COA	Pre-op	16	9	2
	6 Month post-op	9	6	0
	1 Year post-op	0	0	0
	2 Year post-op	0	2	0
ASD + PDA	Pre-op	3	1	1
	6 Month post-op	2	1	1
	1 Year post-op	0	1	0
	2 Year post-op	0	3	0
VSD + PDA	Pre-op	5	2	0
	6 Month post-op	2	0	0
	1 Year post-op	0	0	0
	2 Year post-op	0	0	0
Cyanotic	Pre-op	39	46	8
	6 Month post-op	27	27	5
	1 Year post-op	14	12	4
	2 Year post-op	6	12	2
TOF	Pre-op	10	11	1
	6 Month post-op	9	8	3
	1 Year post-op	2	2	2
	2 Year post-op	0	1	1
Pulmonary atresia	Pre-op	10	8	2
	6 Month post-op	7	4	0
	1 Year post-op	4	2	0
	2 Year post-op	2	4	0
Tricuspid atresia	Pre-op	8	13	3
	6 Month post-op	4	6	0
	1 Year post-op	4	2	1
	2 Year post-op	2	3	0
Single ventricle	Pre-op	11	14	2
	6 Month post-op	7	9	2
	1 Year post-op	4	6	1
	2 Year post-op	2	4	1

CHD, congenital heart disease; WGD, weight growth disorder; HGD, height growth disorder; HCGD, head circumference growth disorder; ASD, atrial septal defect; VSD, ventricular septal defect; PDA, patent ductus arteriosus; COA, coarctation of the aorta; TOF, tetralogy of Fallot.

The highest prevalence of weight, height, and head circumference growth disorders is observed in the 1–5 months age group. As indicated in Table 4, in this age group, 38 individuals (44.7%) had weight below the 5th percentile, 36 individuals (42.35%) had height below the 5th percentile, and 7 individuals (8.23%) had head circumference below the 5th percentile.

**Table 4:** Prevalence of growth disordes among patients within each age groups during the initial preoperative visit.

Age range	Number of patients (%)	Weight below the 5th percentile (%)	Height below the 5th percentile (%)	Head circumference below the 5th percentile (%)
Between 1 and 5 months	85 (42.5)	38 (44.70)	36 (42.35)	7 (8.23)
Between 6 and 11 months	42 (21.0)	23 (54.76)	18 (42.85)	2 (4.76)
Between 12 and 24 months	35 (17.5)	9 (25.71)	15 (42.85)	1 (2.85)
Between 2 and 4 years	17 (8.5)	6 (35.29)	4 (23.53)	1 (5.88)
Greater than 4 years	21 (10.5)	12 (57.14)	8 (38.09)	2 (9.52)

### Six months post-op visit

In 6 months after surgery, the prevalence of the WGD was reduced to 54 patients (27%) and was evenly distributed between cyanotic (27, 50%) and non-cyanotic (27, 50%). HGD was present in 47 patients (23.5%) and in the cyanotic group (27, 57.4%) was more than non-cyanotic group (20, 42.6%). The prevalence of HCGD was also reduced to 7 individuals (3.5%), with 5 cases (71.4%) in the cyanotic and 2 (28.6%) in the non-cyanotic group (Table 1).

According to the logistic regression model adjusted for age and gender, the overall odds of growth disorders were significantly lower compared to the preoperative period. However, analysis of the interaction effects (group × study visit) revealed no significant difference between cyanotic and non-cyanotic patients in the improvement of WGD (OR = 1.032, *p* = .923), HGD (OR = 1.167, *p* = .603), or HCGD (OR = 1.298, *p* = .538), suggesting that both groups had similar reductions in growth disorders by this point (Table 2).

As observed in Table 3, the highest prevalence of weight growth disorders in the 6-month follow-up after surgery was found in patients diagnosed with TOF and COA, each with 9 children (16.66%). Additionally, the highest prevalence of height growth disorders in 9 children (19.14%) was diagnosed with Single Ventricle.

### One-year post-op visit

One year after surgery, the prevalence of WGD was reduced to 18 patients (9%) and was higher in cyanotic patients (14, 77.8%) than non-cyanotic patients (4, 22.2%). HGD was seen in 16 patients (8%) and its prevalence in cyanotic patients (12, 75%) was higher than non-cyanotic patients (4, 25%). Also, the prevalence of HCGD reached 5 individuals (2.5%), with 4 cases (80%) in cyanotic patients and 1 (20%) in non-cyanotic patients (Table 1).

According to the logistic regression model adjusted for age and gender, 1 year after surgery, there was a further significant decrease in the odds of all growth disorders compared to the preoperative period. However, the interaction effects indicated that cyanotic patients had significantly higher odds of WGD (OR = 1.80, 95% CI: 1.15–2.81, *p* = .024) and HGD (OR = 1.89, 95% CI: 1.03–3.49, *p* = .038) compared to non-cyanotic patients at this time. No significant difference was found between groups for HCGD (*p* = .284), suggesting that cyanotic disease was linked to less improvement in weight and height measures 1-year post-surgery (Table 2).

According to Table 3, children diagnosed with Tricuspid Atresia, Pulmonary Atresia, and Single Ventricle had the highest prevalence of weight growth disorders, with 4 cases each (22.22%). Children diagnosed with Single Ventricle had the highest prevalence of height growth disorders, with 6 cases (37.50%) observed in the 1-year follow-up after surgery.

### Two years post-op visit

In evaluation 2 years after surgery, the prevalence of WGD was reduced to 7 (3.5%), which was seen in 6 (85.7%) of cyanotic patients and 1 (14.3%) of non-cyanotic patients. The prevalence of the HGD was 19 patients (9.5%) in the total population, of which 12 (63.2%) was observed in the cyanotic group and 7 (36.8%) in the non-cyanotic group. HCGD was reported in 3 patients (1.5%), of which 2 (66.7%) were in cyanotic patients and 1 (33.3%) were in non-cyanotic (Table 1).

According to the logistic regression model adjusted for age and gender, the overall odds of growth disorders had decreased even further compared to preoperative level. Although the interaction effects suggested that cyanotic patients might have had higher odds of all three growth disorders compared to non-cyanotic patients, none of these differences reached statistical significance: WGD (OR = 1.69, *p* = .109), HGD (OR = 1.57, *p* = .202), and HCGD (OR = 1.20, *p* = .645), (Table 2).

### Surgical and hospitalized duration

The average duration of cardiopulmonary bypass (CPB) in cyanotic patients was 78.14 (±4.02) min, while in non-cyanotic patients, it was 94.34 (±5.17) min. Thirty-one cases of cyanotic patients and 10 cases of non-cyanotic patients were readmitted to the hospital for further diagnostic and therapeutic interventions post-surgery. It is noteworthy that patients diagnosed with Single Ventricle had the highest rate of readmission with 11 cases (26.83%), followed by Tricuspid Atresia with 10 cases (24.39%).

In accordance with the information provided in Table 5, the mean ± SD duration of stay in the Pediatric Intensive Care Unit (PICU) for patients with cyanotic heart disease and growth disorders was 8.11 (±5.47) days, while for non-cyanotic patients, it was 8.84 (±5.83) days. The average duration of hospital stay in the ward was 4.40 (±3.46) days for cyanotic patients and 3.57 (±2.20) days for non-cyanotic patients.

**Table 5:** The mean ± SD length of stay in the PICU and ward for patients with cyanotic and non-cyanotic heart disease and growth disorders.

Type of CHD	Length of stay in the PICU			Length of stay in the ward		
	Total number	All patients	Patients with growth disorders	Total number	All patients	Patients with growth disorders
Cyanotic	854	8.70 (5.11)	8.11 (5.47)	481	5.01 (4.23)	4.40 (3.46)
Non-cyanotic	787	7.87 (5.27)	8.84 (5.83)	389	4.18 (3.57)	3.57 (2.20)

### Complications

Thirty-seven individuals (18.5%) among children with congenital heart disease experienced postoperative complications, with 4 cases of neurological, 7 cases of renal, and 11 cases of gastrointestinal complications in cyanotic patients. In non-cyanotic patients, there were 2 cases of neurological, 5 cases of renal, and 8 cases of gastrointestinal complications. Based on the information outlined in Table 5, the postoperative mortality rate for cyanotic and non-cyanotic patients was 23 cases and 7 cases, respectively. Among the deceased individuals during the last visit, 15 cases (50%) had weight below 5th percentile, and 4 cases (13.33%) had weight between 5th and 10th percentile. Notably, 12 children (52.17%) diagnosed with cyanotic heart disease and 3 children (42.85%) diagnosed with non-cyanotic heart disease had growth disorders and died after surgery (Table 6). Furthermore, the highest mortality rate associated with growth disorders was observed in children with Pulmonary Atresia, with 4 cases (26.66%).

**Table 6:** The association between growth disorders and mortality in children with congenital heart disease after surgical intervention.

Type of CHD	The total number of mortalities	Weight below the 5th percentile (%)	Height below the 5th percentile (%)	Head circumference below the 5th percentile (%)
Cyanotic	23	12 (52.17)	9 (39.13)	4 (17.39)
Non-cyanotic	7	3 (42.85)	4 (57.14)	2 (28.57)

## Discussion

Children diagnosed with CHD face a notable risk of developmental delays and disorders. While many of these children may not encounter significant issues in their early years, challenges often emerge in the second year of life due to the escalating severity of the disease. This escalation impedes weight gain and hampers various aspects of their intellectual development (12). Advancements in diagnostic techniques, interventional procedures, surgical methods, and specialized pediatric cardiac intensive care have contributed to enhanced long-term survival rates among children diagnosed with CHDs (13).

Approximately 50 years ago, before the advent of pediatric cardiac surgery, only around 30% of children with CHD survived to reach adulthood. Today, with advancements in surgical techniques and comprehensive postoperative care, coupled with diagnostic tools like echocardiography and fetal scanning, it is now estimated that about 85% of children diagnosed with CHD can expect to live into adulthood. Growth retardation emerges as a common occurrence in infants with CHD. Accurate growth charts, depicting height and weight in both absolute values and percentiles, play a crucial role in the initial assessment and ongoing monitoring of children facing significant cardiac challenges (12, 14).

In our investigation, a notable percentage (24.5%) of newborns experienced weight growth issues, with 18% facing height growth challenges and 5.5% exhibiting head circumference growth disorders. This mirrors findings from a study by Diao et al. (15), indicating higher prevalence rates of preoperative underweight, stunting, and wasting in children with CHD compared to healthy counterparts. The results underscore the early emergence of growth issues in CHD patients, emphasizing the crucial role of timely detection for effective intervention.

Malnutrition in children with CHD stems from various factors like reduced energy intake due to poor appetite, intestinal dysfunction leading to nutrient absorption decline, complications like heart failure affecting cardiac output and nutrient utilization, and increased myocardial contraction burden due to structural abnormalities (15). In our study, a cross follow-ups at 6 months, 1 year, and 2 years post-operation, substantial enhancements in children's growth disorders were evident. Earlier studies have corroborated that children undergoing corrective cardiovascular surgery may demonstrate a recovery in both height and weight (16), consistent with the observations from our study.

In a case-control investigation conducted by Noori et al. in Zahedan, Iran, the growth status of children with CHD, both cyanotic and non-cyanotic, with and without pulmonary hypertension, was assessed in comparison to that of healthy children. The study revealed that CHD children exhibited significantly lower weight and head circumference compared to their healthy counterparts. Furthermore, CHD patients without surgical intervention demonstrated reduced weight, height, and head circumference intake in comparison to CHD patients who underwent surgery (17).

Another study by Nuralim et al. (12), which involved 40 patients with non-cyanotic CHD who underwent heart repair procedures, emphasized the importance of early intervention for CHD in preventing cardiac growth distortion. The study revealed a significant impact of repair surgery on growth 2–3 years post-operation (*p*-value of 0.016). This aligns with Li et al.'s (18) earlier study, indicating immediate growth improvement after successful surgery in patients under 12 months.

A retrospective study involving 331 infants with CHD undergoing cardiac surgery before the age of one reveals enduring growth failure and malnutrition post-surgery. 16% displayed growth failure (<−2SD), and 14% exhibited a <−2SD weight/height ratio at 6–12 months. Perinatal factors (prenatal diagnosis, <3 kg birth weight, genetic syndrome, complex CHD) were linked to prolonged growth failure. The study highlights that delayed surgery (>30 days) is associated with an elevated risk of malnutrition. Subsequent findings suggest that infants with non-operated heart disease, developing pulmonary overload and congestive heart failure weeks after surgery, face a negative impact on weight gain due to fluid restriction and diuretics (19).

The systematic review and meta-analysis conducted by Van den Eynde et al. tracked the trajectory of *z* scores for height and weight in single ventricle patients undergoing corrective surgery, specifically the Glenn-Fontan procedure, and their long-term follow-up. Results from 19 studies encompassing 2006 participants revealed a significant decrease in *z* scores for height and weight from birth to the interstage period. However, approximately 50% recovery was observed after the Glenn procedure. Beyond 10 years post-Fontan procedure, weight *z* scores exhibited normalization despite persistently lower height, leading to an increased body mass index (20).

Ni et al. conducted a systematic review and meta-analysis aiming to assess the impact of high-energy and/or high-protein enteral nutrition (EN) in post-surgery children with CHD. The analysis, encompassing nine studies with 609 children, indicates that implementing high-energy or high-protein EN in infants after CHD surgery doesn't escalate the occurrence of feeding intolerance or affect fluid intake. Notably, the intervention results in increased weight and weight-for-age *z*-score (WAZ) while concurrently reducing the duration of mechanical ventilation, length of ICU stay, and overall hospital stay (21).

Postoperative complications were observed in 18.5% of cases, with neurological, renal, and gastrointestinal complications exhibiting notable rates. The study reports a postoperative mortality rate of 23 cases in cyanotic patients and 7 cases in non-cyanotic patients. Among the deceased, 50% had a weight below the 5th percentile and 13.33% between the 5th and 10th percentiles. In a retrospective analysis, Javed et al. (22) reported that the predominant complications included extended postoperative mechanical ventilation (27%), pleural effusion (21%), excessive bleeding (19%), cardiac arrest (18%), and systemic infections (18%). Zeng et al. (23) noted that patients who were younger, lighter, or had cyanotic lesions experienced higher complications. According to a retrospective observational study conducted by Agarwal et al. (24), postoperative complications in pediatric cardiac surgery were linked to extended periods of mechanical ventilation, prolonged hospital stays, and heightened mortality rates. Mortality, particularly associated with growth disorders, underscores the profound impact of these challenges on overall health outcomes. This finding is particularly poignant, highlighting the urgent need for targeted interventions to mitigate mortality risks associated with growth impairments.

## Conclusion

In conclusion, our study underscores the profound impact of congenital heart disease on children's growth, emphasizing the critical need for tailored interventions. This approach not only alleviates the financial burden on both the community and families but also plays a pivotal role in diminishing mortality and complications associated with CHD. The integration of timely interventions in the realm of growth and nutrition stands as a crucial step towards optimizing the overall well-being of these young patients.

## Data Availability

The raw data supporting the conclusions of this article will be made available by the authors, without undue reservation.
